# Spontaneous light-induced Turing patterns in a dye-doped twisted nematic layer

**DOI:** 10.1038/s41598-018-31206-x

**Published:** 2018-08-27

**Authors:** Ignacio Andrade-Silva, Umberto Bortolozzo, Marcel G. Clerc, Gregorio González-Cortés, Stefania Residori, Mario Wilson

**Affiliations:** 1 Departamento de Física and Millennium Institute for Research in Optics, Facultad de Ciencias Físicas y Matemáticas, Universidad de Chile, Casilla, 487-3 Santiago, Chile; 2 Institut de Physique de Nice, UMR 7010, Université de Nice-Sophia Antipolis, CNRS, 1361 Route des Lucioles, 06560 Valbonne, France; 3 CONACYT – CICESE, Carretera Ensenada-Tijuana 3918, Zona Playitas, C.P. 22860 Ensenada, Mexico

## Abstract

Optical pattern formation is usually due either to the combination of diffraction and nonlinearity in a Kerr medium or to the temporal modulation of light in a photosensitive chemical reaction. Here, we show a different mechanism by which light spontaneously induces stripe domains between nematic states in a twisted nematic liquid crystal layer doped with azo-dyes. Thanks to the photoisomerization process of the dopants, light in the absorption band of the dopants creates spontaneous patterns without the need of temporal modulation, diffraction, Kerr or other optical nonlinearity, but based on the different scales for dopant transport processes and nematic order parameter, which identifies a genuine Turing mechanism for this instability. Theoretically, the emergence of the stripe patterns is described on the basis of a model for the dopant concentration coupled with the nematic order parameter.

## Introduction

**Figure 1:**
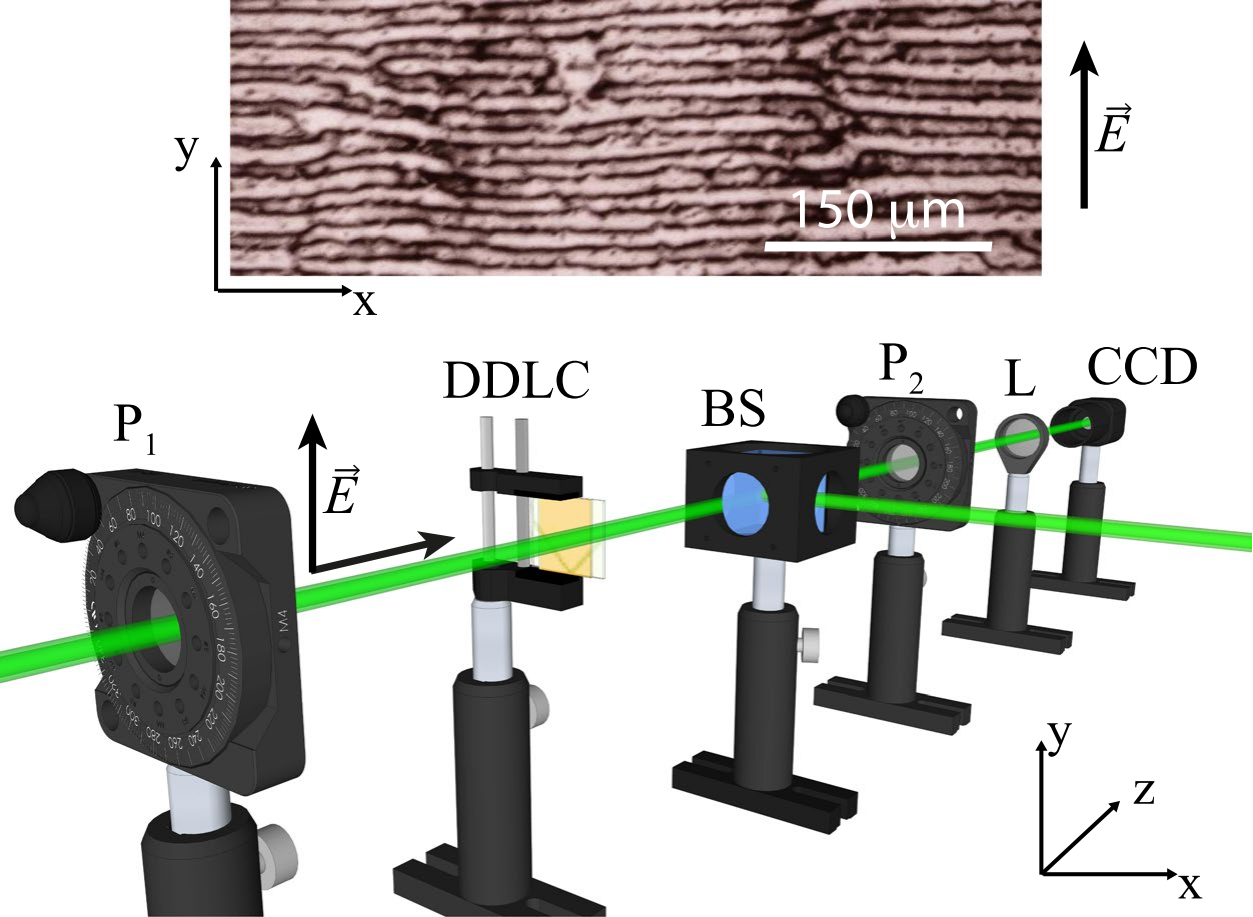
Schematic of the experimental setup. *P*_1_ and *P*_2_: polarizers along *y* and *x* axis, respectively, *L*: plano-convex lens, DDLC: dye-doped twisted liquid crystal cell (E7 with 0.75% in weight of Methyl-Red), BS: beam-splitter, and CCD: charge-coupled device camera. The system is irradiated by a frequency doubled Nd^+3^:YVO_4_ laser (*λ*_0_ = 532 *nm*) with vertical polarization (*y*-axis). The upper panel accounts for the typical stripe domain observed near to the center of the illuminated region.

Non-equilibrium processes often lead to the formation of spatial periodic structures developed from a homogeneous state through the spontaneous breaking of symmetries^1–4^. This self-organization usually is a consequence of the force imbalance or transport optimization of energy, momenta and/or particles. Initially, these patterns were understood as the saturation of linear normal modes using nonlinear effects^3,4^. Hence, the characteristic length of these patterns is determined by the geometrical dimensions of the system under study. Classic examples are the Benard and Taylor-Couette patterns^4^. Another mechanism proposed to understand the pattern formation is based on the difference in transport or coupling processes in chemical reactions, known as *Turing instability*^5^. These patterns, *Turing patterns*, are characterised by having an intrinsic characteristic length, which is not determined by external factors or geometrical dimensions but by the diffusive coefficients and temporal scales of the system under study. This mechanism has been applied from biology to optics, passing through chemistry and physics^2,4,6,7^. An example of pattern formation in liquid crystals is the electroconvection^8^, which results in charge transport and convection effects. Similar patterns are observed in thin liquid crystal layers close to nematic-smectic transition^9–11^, thin hybrid nematic layer^12^ and polymer nematic liquid crystal^13^, where one of the elastic constants is much larger than the other ones. In the case of light and matter interactions, the spontaneous transverse optical grating formation has been observed in slightly asymmetric single-feedback mirror experiments using nematic liquid crystals as nonlinear optical media^14^. These patterns come from the interference between counter-propagating light in a nonlinear medium, that is, diffraction transforms phase variations into amplitude modulations while the optical nonlinearity converts amplitude into phase modulations. Likewise, patterns have also been observed by temporal modulation of light in photosensitive chemical reactions^15^. Light-induced effects in absorbing liquid crystals attracted a considerable interest for their potential applications in optical switching and image storage^16^. There are a number of phenomena connected with excitations of liquid crystal molecules or of dye-dopants added to a liquid crystal matrix host. Photoinduced conformational transformations, such as trans-cis isomerization of azo compounds, can change the orientational order parameter (see textbook^16^ and reference therein), influence the chiral properties^17^, or induce phase transitions^18,19^. In this letter, we show that stripe domain patterns between different nematic states (i.e. molecules that are locally alternated between regions of higher and lower orientational order) can spontaneously arise in a dye-doped twisted nematic liquid crystal layer when illuminated under appropriate conditions. In this case, the pattern formation is not mediated by the light diffraction or the temporal light modulation, neither by the Kerr or other optical nonlinearity, but originates from the different scales for dopant concentration and order parameter transport process, hence, identifying a novel mechanism of light-induced Turing instability. Experimentally, we consider a twisted nematic liquid crystal cell, namely, the liquid crystal molecules have mutually orthogonal planar anchoring onto the two glass substrates that constitute the confining walls of the cell. When the liquid crystal sample is illuminated with a linearly polarized Gaussian beam at a wavelength inside the absorption band of the dopants, for a certain critical input power value, a transition from the homogeneous nematic state to a spatially modulated one with a striped structure is observed (cf. Fig. 1). This structure accounts for a spatial modulation of the nematic liquid crystal molecular orientation. Noticeably, the orientation of the stripes is perpendicular to the linear polarization of the illuminating light. Theoretically, we are able to describe the emergence of the stripe patterns on the basis of a model for the concentration of azo-dye dopants in the excited state (*cis*-state) coupled with the order parameter of the twisted nematic layer. This model allows us to identify the mechanism of pattern emergence, which is due to the different scales for transport processes of dopants and order parameter, i.e. it corresponds to a Turing instability. Adiabatically, by eliminating the dopant concentration, the order parameter fulfills a Turing-Swift-Hohenberg type equation, which allows us to determine analytically the instability criterion. The Turing-Swift-Hohenberg equation is a paradigmatic model for pattern formation in several contexts, such as hydrodynamics, chemistry, plant ecology, nonlinear optics, and elastic materials^4^.

## Results

### Light-induced pattern formation

Applying a light beam with wavelength in the absorption band of the methyl red^20^ creates the gradual emergence of stripe domains between nematic states, first in the central region of the illuminated area, then, the domains invade the whole illuminated zone. This process takes about three hours to invade an area of 3 *cm*^2^. After a while the different domains are merged generating several defects; mainly dislocations are identified, which are characterized by joints, that is, locally, regions with different wavenumbers^3^. Figure 1 shows the typical stripe patterns with several dislocations observed. It is also observed that if the sample is illuminated with a wavelength which is not in the absorption band of the dye, the pattern does not emerge. In particular, samples of dye-doped liquid crystal with identical configurations illuminated with red light (He-Ne Laser @ *λ* = 633 nm and *I* = 35 mW) at similar power do not exhibit any effect.

Noticeably, the patterns are mostly oriented orthogonally to the direction of the light electric field. Indeed, when the electric field is rotated the patterns are reoriented in the direction orthogonal to it. Notice that the pattern wavelength (around 35 *μ*m) does not correspond to the cell thickness, but it is on the same order of magnitude.

**Figure 2:**
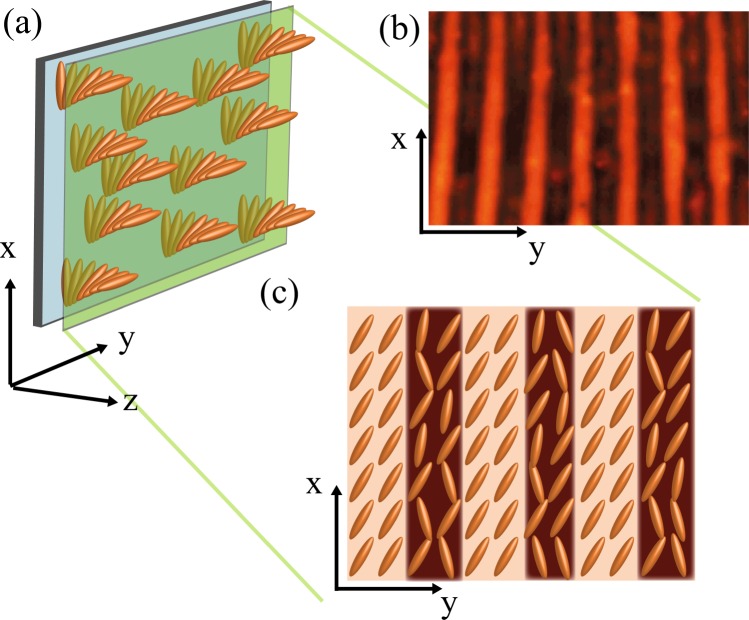
Stripe domain induced by photo-isomerization in a dye-doped twisted nematic liquid crystal cell. (**a**) Schematic representation of the twisted molecular configuration for a small portion of the unenlightened cell. (**b**) Experimental stripe domain induced by photo-isomerization of the dyes. (**c**) Schematic representation of an intermediate plane of the cell of the molecular configuration for a small portion of the enlightened cell.

The light intensity emerging from the dye-doped nematic liquid crystal sample accounts for the molecular orientation, which is characterized by transversal spatial oscillations. Fig. 2(c) schematizes the molecular rearrangement in an intermediate plane of the sample.

As the sample is not illuminated, the dye molecules are in the *trans* state and oriented along the liquid crystal nematic director^21^. When the sample is illuminated by a light beam in the absorption band of the dopants, the scenario changes because the light induces a photo-isomerization process and the dopants undergo a transition from the *trans* to the *cis* state, corresponding to different molecular configurations of the azo-dyes^16,21^. Likewise, the dyes in the *cis* state produce the reorientation of the liquid crystal molecules. However, the liquid crystal molecules are oriented in different directions. Hence, this transition is characterised by a decrement of the liquid crystal molecular order–which corresponds to an entropic effect–and, correspondingly, by the modification of the average refractive index $\bar{n}$ of the sample^21^, which is averaged along the longitudinal direction *z*, so that the average refractive index $\bar{n}$ remains a function of the cross-sectional coordinates (*x*, *y*). This index has the form^16,22^

1 $$\bar{n}(x,y,z)=\frac{1}{d}{\int }_{0}^{d}\,\frac{{n}_{0}{n}_{e}}{\sqrt{{n}_{o}^{2}{\cos }^{2}\theta (x,y,z)+{n}_{e}^{2}{\sin }^{2}\theta (x,y,z)}}dz,$$

where *d* is the thickness of the sample, *n*_0_ and *n*_*e*_ are the ordinary and extraordinary refractive index of the liquid crystal respectively, and *θ*(*x*, *y*, *z*) is the average angle between the molecules and the horizontal axis of the sample. For example, if the molecules are oriented in the plane of the sample *θ* = 0 and $\bar{n}$ = *n*_*e*_. Indeed, modifications of molecular order produce a change in $\bar{n}$. Note that clearer stripes (darker stripes) in Figs 1 and 2(b) account for regions where molecules are more ordered (disordered).

**Figure 3:**
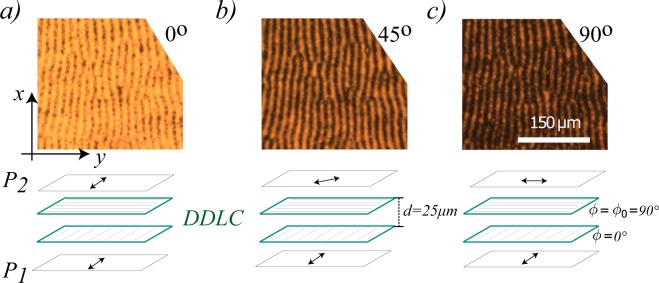
Stripe domain induced by photo-isomerization in a dye-doped twisted nematic liquid crystal cell between polarizers *P*_1_ and *P*_2_ with different relative angles. The upper and bottom panels, respectively, account for the observed snapshots and schematic configuration of the polarizers and liquid crystal sample. (**a**) Both polarizer are parallel, the relative angle between polarizers is 0°, (**b**) the relative angle between polarizers is 45°, and (**c**) both polarizers are crossed, that is, the relative angle between polarizers is 90°.

To characterize the molecular orientational configurational structure of the observed patterns, we have analyzed the sample by rotating *P*_2_ polarizer (analyzer) concerning to *P*_1_ polarizer, which is a standard technique for characterizing liquid crystal textures^23^. When the dye-doped twisted liquid crystal sample is between two parallel polarizers, that is, the angle between polarizers is 0°, the light that crosses the sample exhibits a striped pattern with a different tenuous intensity. Fig. 3(a) shows the typical picture observed in this configuration of the polarizers. The stripes and between stripes have different colors. Hence, one infers that these regions have a different average refractive index. When the analyzer is rotated with respect to the first polarizer, the image darkens slightly and the patterns continue to be observed (see Fig. 3(b)). When the polarizers are orthogonal, the angle between the analyzer and the first polarizer is 90°; one continues to observe the pattern with darker browns (cf. Fig. 3(c)). This analysis is a rigorous proof that the material is birefringent. Note that the stripes are brown and not black. Indeed, the light crosses these regions. Therefore, both the stripes and between stripes the material are liquid crystal phase with a different average refractive index. This index is spatially modulated.

### Theoretical description

To describe the dynamics of the twisted nematic liquid crystal layer, one can introduce a scalar order parameter *S*($\overrightarrow{r},t$) that accounts for the alignment of the molecules along the director **n** = (sin(*ϕ*), cos(*ϕ*), 0)^8,24^, defined by $S\equiv 3\langle {\cos }^{2}\,\theta \rangle \mathrm{/2}-\mathrm{1/2}$ where the brackets 〈⋅〉 mean spatial average in a microscopic element volume and *θ* is the angle between the molecules and the director **n**^8^. Due to the anchoring conditions, the director **n** is contained in the transversal plane to the direction of light propagation. Indeed, the director is characterized by the *ϕ* angle defined concerning the axis of the *x* coordinate. Hence, the scalar order parameter for a perfectly aligned nematic phase is *S* = 1 and for an isotropic phase is *S* = 0. Namely, different values of S account for nematic states with different molecular order. Based on the Landau-de Gennes and Ericksen theory, the transition between a nematic state and isotropic liquid in a twisted nematic liquid crystal layer is described by the dimensionless equation^8,23,24^

2 $${\partial }_{t}S(\overrightarrow{r},t)=-\,\tilde{A}S+B{S}^{2}-E{S}^{3}+d{\overrightarrow{\nabla }}^{2}S-DS{(\overrightarrow{\nabla }\varphi )}^{2},$$

3 $$S{\partial }_{t}\varphi (\overrightarrow{r},t)=DS{\overrightarrow{\nabla }}^{2}\varphi +2D\overrightarrow{\nabla }S\cdot \overrightarrow{\nabla }\varphi ,$$

where *Ã*, *B* and *E* are parameters that characterize this transition and *D* stands for the elastic coupling (see the textbook^23^ for details of the parameters). A twisted cell of thickness *h* is characterised by the boundary conditions *ϕ*(*x*, *y*, *z* = 0, *t*) = 0, *ϕ*(*x*, *y*, *z* = *d*, *t*) = *ϕ*_0_ and *d* is the thickness of the liquid crystal layer (see Fig. 3). The cell that we have considered in our experiment is characterised by having *ϕ*_0_ = *π*/2. To describe our system, we consider that the liquid crystal cell is sufficiently thin and that the director rotates uniformly from one plate to the other. Under these assumptions, we get *S*($\overrightarrow{r},t$) = *S*(*x*, *y*, *t*) and *ϕ*($\overrightarrow{r},t$) = *πz*/2*h*. Note that this solution trivially satisfies Eqs (2) and (3) is rewritten as

4 $${\partial }_{t}S(x,y,t)=-\,AS+B{S}^{2}-E{S}^{3}+D{\overrightarrow{\nabla }}_{\perp }^{2}S,$$

where *A* ≡ *Ã* + *D*(*π*/2*h*)^2^ and ${\overrightarrow{\nabla }}_{\perp }^{2}$ stands for the laplacian in transversal coordinate. This model predicts that the nematic and the isotropic liquid transition in a twisted cell is of subcritical nature. It is important to mention that the previous model Eq. (4), renormalizing the linear term, also describes planar (*ϕ*($\overrightarrow{r},t$) = *ϕ*_0_ constant) and homeotropic (**n** = $\hat{z}$) cells^8^. On the other hand, the concentration of molecules in the *cis*-state *C*($\overrightarrow{r},t$) in a thin layer satisfies a relaxation and diffusion equation of the form^21^

5 $${\partial }_{t}C=-\,\lambda [C-{C}_{0}(I)]+\delta {\overrightarrow{\nabla }}_{\perp }^{2}C,$$

where *λ* is the decay rate related to the transition from *cis* to *trans* state by thermal relaxation. *C*_0_ is the equilibrium concentration of molecules in the *cis* state that is proportional to the total intensity of the incident light *I*. Precisely, *C*_0_(*I*) ≡ *γI*/(1 + *ηI*) where *γ* and *η* are dimensional parameters^21^. *δ* is the diffusion coefficient of the concentration of *cis* state.

The inclusion of dye-dopants increases the nonlinear response of liquid crystals under the excitation of external fields^16–19,21,25^. Indeed, the behavior of liquid crystals changes drastically when dopants are considered. To describe the pattern formation induced by the photo-isomerization process in a dye-doped twisted nematic layer, let us consider the concentration of molecules in the *cis*-state *C*(${\overrightarrow{r}}_{\perp },t$) and the scalar order parameter *S*(${\overrightarrow{r}}_{\perp },t$), which satisfy the rate equations

6 $$\begin{array}{rcl}{\partial }_{t}C & = & -\lambda [C-{C}_{0}(I)+\alpha S]+{\delta }_{\Vert }{\partial }_{xx}C+{\delta }_{\perp }{\partial }_{yy}C+D{\overrightarrow{\nabla }}_{\perp }^{2}S,\\ {\partial }_{t}S & = & -(A+\beta C)S+B{S}^{2}-{S}^{3}+{\overrightarrow{\nabla }}_{\perp }^{2}S+D{\overrightarrow{\nabla }}_{\perp }^{2}C,\end{array}$$

**Figure 4:**
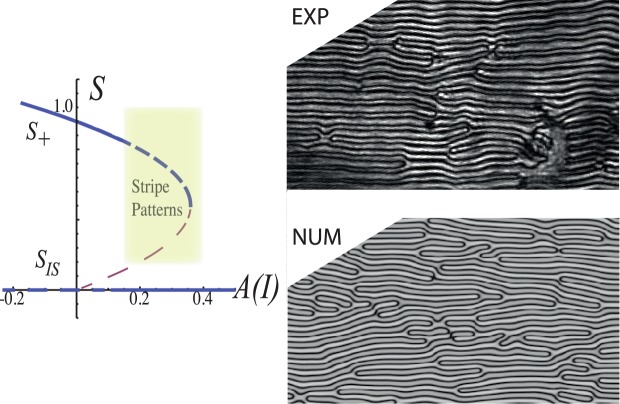
Comparison of experimental (top right panel) and numerical (bottom right panel) stripe domains induced by photoisomerization in the dye-doped twisted nematic liquid crystal layer. Represented numerical simulations of Eq. (6) were done with *a* = 0.24, *β* = 0.05, *b* = 1, *e* = 1, *d* = 1, *D* = 0.5, *C*_0_ = 0.01, *λ* = 1, *δ*_||_ = 0.8, *δ*_⊥_ = 1 and *α* = 4. Left panel accounts for the bifurcation diagram as function of *A*(*I*) with the same other parameters. Continuos and dashed curves stand for stable and unstable state, respectively.

${\overrightarrow{r}}_{\perp }$ = {*x*, *y*} stands for the transversal coordinate of the layer, *α* accounts for the reduction of cis-state concentration when the liquid crystal molecules are more aligned (larger *S*) because the dopants tend to be oriented in the direction of the molecules (transition from *cis* to *trans*)^21^. Due to the process of photo-isomerization and elastic features of the liquid crystal, all transport processes must be anisotropic^8,16,21^. Indeed, {*δ*_⊥_, *δ*_||_} are the diffusion coefficients of the dopant concentration in the parallel and orthogonal direction with respect to the incident light electric field. *β* stands for the entropic effect of the photo-isomerization process, that is, by increasing the concentration of the *cis* molecules the disordered state is favoured. Finally, *D* accounts for the mutual transport process. Note that a gradient in dopant concentration induces propagation of the order parameter^26^. In addition, for simplicity, we only consider anisotropy in the diffusion of dopant. Notice model, Eq. (6), is a non-variational model, namely, this set of equations do not come from the variation of a free energy. This is because the system is out of equilibrium and the forcing is mediated by permanent light^2^. For small *α* and intensity *I*, the cis-state concentration satisfies *C* = *C*_0_(*I*) ≈ *γI*. Hence, the parameter order *S* satisfies the Landau-De-Gennes model for the nematic to isotropic transition induced by photo-isomerization^25^, namely, the bifurcation parameter *A*(*I*) ≡ *A* + *βγI* is controlled by the light intensity. In this case, if the intensity has a Gaussian profile, the light can induce front propagation from the isotropic (*S*_*IS*_) to the nematic phase (*S*_+_)^25^. The above model, Eq. (6), has two homogeneous states (*S*, *C*) = (*S*_*IS*_, *C*_*IS*_) ≡ (0, *C*_0_) and $(S,C)=({S}_{\pm },{C}_{\pm })\equiv ([\alpha \beta +B\pm \sqrt{{(\alpha \beta +B)}^{2}-\mathrm{4(}A+\beta {C}_{0})}]/\mathrm{2,}\,{C}_{0}-\alpha {S}_{\pm })$ that account, respectively, for an isotropic liquid and a dye-doped nematic phase. For small coupling *α* between the order parameter and the *cis* concentration, the stable nematic phase corresponds to (*S*_+_, *C*_+_). By increasing the coupling parameter, the homogeneous nematic phase becomes unstable, giving rise to the emergence of striped domains. Namely, the order parameter *S* exhibits spatial modulation. Figure 4 shows the typical observed pattern when a weak anisotropy is considered (*δ*_||_ ~ *δ*_⊥_) and the associated bifurcation diagram. Note that the texture observed experimentally shows quite a good agreement with that observed from model Eq. (6). Hence, the order parameter *S* and concentration of dopants *c* exhibit spatial oscillations along the direction of the light electric field. This physically means that molecules locally alternate between regions of higher and lower orientational order, as depicted in Fig. 2(c). Due to the refractive index dependence with the molecular orientation, the light that crosses the liquid crystal cell displays bands of different intensities as seen in Figs 1, 2(b) and 4.

To figure out the physical mechanism giving rise to the emergence of the stripe domains, a linear stability analysis over the homogeneous nematic phase (*S*_+_, *c*_+_) was performed. We first consider, for the sake of simplicity, the isotropic case of model (6), i.e. *δ*_||_ = *δ*_⊥_ = *δ*. By using a perturbation of the form $(S,C)=({S}_{+},{C}_{+})+(\delta s,\delta c){e}^{i\overrightarrow{k}\cdot {\overrightarrow{r}}_{\perp }+\sigma t}$, where *σ* is the growth rate and $\overrightarrow{k}$ the wavenumber vector, in Eq. (6), and by keeping only the linear terms, we obtain a relation between the growth rate and wavenumber vector, *σ*($\overrightarrow{k}$; {*λ*, *C*_0_, *δ*, *α*, *D*, *A*}), which is a complex function of two components.

**Figure 5:**
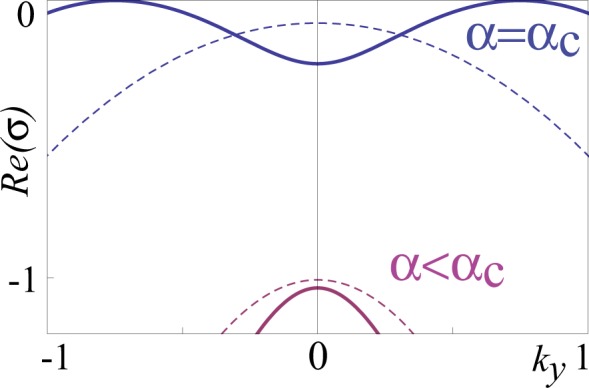
Growth rate *Re*(*σ*) of dye-doped nematics phase (*S*_+_, *C*_+_) as a function of the wavenumber in *y*-direction *k*_*y*_ for different value of coupling *α* parameter and the same other previous parameters. The blue and purple curves correspond to different components of the real part of the growth rate *σ*. The solid and dashed curves correspond, respectively, to the growth rate at critical *α* ≡ *α*_*c*_ = 4.1 (spatial instability) and at *α* = 1 < *α*_*c*_ (below the instability).

Figure 5 shows the real part of the growth rate, *Re*(*σ*), as a function of the wavelength modulus $k=\Vert \overrightarrow{k}\Vert$ for fixed parameters at the spatial bifurcation (*α* = *α*_*c*_) and below the spatial instability (*α* < *α*_*c*_). Note that the spatial instability of the homogeneous nematic phase occurs while increasing *α* above a critical value *α*_*c*_. We then consider the anisotropic case (*δ*_||_ ≠ *δ*_⊥_) of model (6). In this case, the instability occurs in the most unstable direction on wavenumber space, which depends on the relative values of *δ*_|_ and *δ*_⊥_, so that, beyond the instability the stripes will be along the direction of the smallest diffusion coefficient, which corresponds to the direction orthogonal to the light electric field (*δ*_||_ > *δ*_⊥_). Hence, we can conclude that the differences of scales of transport and relaxation processes for the order parameter *S*($\overrightarrow{r},t$) and the *cis* state concentration *C*($\overrightarrow{r},t$) are responsible for the emergence of patterns.

Due to the complex and long expression of the growth rate *σ* as a function of the parameters, the former study is only accessible through numerical analysis. To understand more deeply and analytically the origin of the spatial instability, we consider the extreme limit in which one variable, the *cis* state concentration, follows adiabatically the order parameter *S*^27^. Indeed, by assuming that the temporal evolution of the *cis* concentration is rapid compared to the dynamics of the order parameter, i.e. $\lambda \gg 1$, and by using Neumann series, one can approach, at dominant order, the *cis* concentration by $C\simeq {C}_{0}(I)-\alpha S+D{\nabla }^{2}S/\lambda -\alpha {\delta }_{\Vert }{\partial }_{xx}S/\lambda -\alpha {\delta }_{\perp }{\partial }_{yy}S/\lambda$. By introducing this expression in the equation for the order parameter, one gets

7 $$\begin{array}{rcl}{\partial }_{t}S & = & -[A+\beta {C}_{0}(I)]S+(B+\alpha \beta ){S}^{2}-{S}^{3}+\frac{{D}^{2}}{\lambda }{\nabla }^{4}S\\  &  & +\,\mathrm{(1}-D\alpha ){\nabla }^{2}S-\frac{\alpha }{\lambda }({\delta }_{\Vert }{\partial }_{xx}+{\delta }_{\perp }{\partial }_{yy}){\nabla }^{2}S\mathrm{.}\end{array}$$

This equation corresponds to a Turing-Swift-Hohenberg type equation^4,28^. This type of model generically describes pattern formation in several contexts, ranging from biology, ecology, chemistry to physics. A necessary condition for the observation of patterns is that the effective diffusion coefficient is negative (anti diffusion), i.e. *αD* < 1, which, indeed, imposes that the system needs two different transport scales in order to observe the emergence of patterns. By increasing *α* the isotropic phase exhibits a spatial instability for $\mathrm{4(}A+\beta {C}_{0})+\mathrm{5(1}+\beta \alpha ){S}_{+}$ = $({D}^{2}-\alpha {\delta }_{\Vert }){k}_{c}^{4}/\lambda -\mathrm{(1}-D\alpha ){k}_{c}^{2}$, where the critical wavenumber is ${k}_{c}$ = $\sqrt{\mathrm{(1}-D\alpha )\lambda \mathrm{/2(}{D}^{2}-\alpha {\delta }_{\Vert })}$.

Figure 5 shows a typical stripe domain obtained from numerical simulation of Eq. (7). All numerical simulations presented are obtained by considering finite differences code with Runge-Kutta order-4 algorithm. Therefore, the simple Turing-Swift-Hohenberg type Eq. (7) qualitatively well describes the dynamics of stripe domains observed in the experiments.

**Figure 6:**
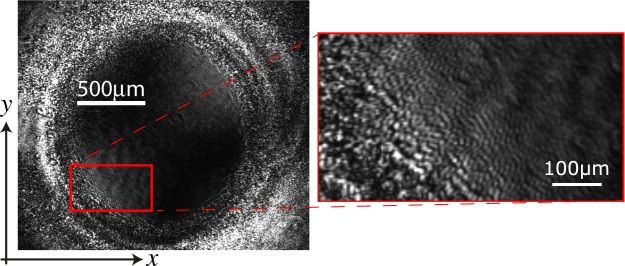
Experimental pattern formation induced by photo-isomerization in a dye-doped nematic liquid crystal cell with planar alignment. Snapshot obtained considering the same experimental configuration as in Fig. 1 by *P* = 900 *mW* and waist of 1.5 mm, changing the dye-doped sample of twisted liquid crystal cell by a planar one, considering the same liquid crystal matrix and dye in a concentration of 1% by weight. The inset shows the typical patterns observed in a dye-doped laminar nematic liquid crystal layer.

The theoretical description presented also contains the case of dye-doped planar nematic liquid crystal cells, considering constant *ϕ*($\overrightarrow{r},t$) = *ϕ*_0_. However, in this case, the effective linear parameter *A* in *S*($\overrightarrow{r},t$) is modified (*A* = *Ã*). Besides, one expects that because the dopants are oriented in a single direction the coupling with the light is weaker, that is, the *C*_0_(*I*) parameter must be smaller. Therefore, one requires higher light intensity to induce patterns. Figure 6 shows experimental pattern induced by photo-isomerization in a dye-doped laminar nematic liquid crystal cell. The wavelength observed is smaller than that found in the twisted configuration. However, these observations allow us to conclude that the phenomenon of spontaneous light-induced patterns in a dye-doped nematic liquid crystal layer is robust and does not depend on the particular choice of the liquid crystal alignment.

## Discussion

In conclusion, we have experimentally shown that linearly polarized light induces stripe domains between nematic states in a twisted dye-doped nematic liquid crystal cell when the intensity is above a critical value. Indeed, light creates spontaneous patterns without the need of temporal modulation, diffraction, Kerr or other optical nonlinearity, but just based on the different scales for dopant transport processes and nematic order parameter, which identifies a genuine Turing mechanism for this instability. Theoretically, we are able to describe the emergence of stripe patterns and to show that the different scales for dopant transport processes and the order parameter are responsible for their emergence. In the limit where there is a significant timescale separation between variables, a simple Turing-Swift-Hohenberg type model is derived, which allows performing an analytical analysis. Experimentally, we observed rich front dynamics between stripe domains and homogenous nematic phase, showing the system’s capability of responding to the optical addressing and opening novel perspectives in the field of optical control of micro-structured soft matter materials and spontaneously self-organized optical media.

## Methods

The nematic liquid crystals are characterised by having a rod-like molecular structure^8,16,23^, that is, these molecules are distinguished by having a uniaxial structure. In a temperature range, these molecules are locally aligned forming the nematic phase (thermotropic liquid crystal)^8,16,23^. To substantially increase the coupling between the light and the nematic liquid crystal dye-dopants are added to a liquid crystal matrix host. Then a requirement is that the dyes-dopant molecules have a uniaxial rod-like structure^16^, which is not necessarily a liquid crystal. Likewise, the concentration in weight of the dye-dopant in the liquid crystal must be low in order to not degrade the properties of the liquid crystal and ensure the solubility of the mixture. In the case of E7 liquid crystal and methyl-red dye, the experiments were performed in mixtures in the range of 0.25% up to 1% concentration by weight.

The experimental setup is depicted in Fig. 1. A dye-doped nematic liquid crystal (DDLC) cell subjected to an orthogonal Gaussian laser beam is studied. The cell was filled with an E7 nematic liquid crystal doped with the azo-dye Methyl-Red at a concentration of 0.75% in weight. The elastic constants of the liquid crystal under consideration are, respectively, *K*_1_ = 11.2, *K*_2_ = 6.8, and *K*_3_ = 18.6 (×10^−12^ N) and the relative parallel and perpendicular dielectric constants are *ε*_||_ = 18.96 and *ε*_⊥_ = 5.16. The cell consists of two glass plates coated with Poly-Vinyl-Alcohol (PVA) and rubbed to favour the planar alignment of the liquid crystal molecules, nematic director parallel to the substrates. The cell is a sandwich type with *d* = 25 *μm* thick spacers. The gap is filled with the dye-doped nematics liquid crystal. The transversal region covered by the liquid crystal is a square of the order of 4 cm^2^. The rubbing directions on the glass plates were such to impose twisted anchoring conditions of the liquid crystal molecules, namely, parallel anchoring directions for the molecules on the confining plates, (see Fig. 2). This type of configuration favours the dopant molecules to be positioned with different orientations, which ensures a relevant coupling with the light that crosses the sample. Figure 2(a) illustrates schematically the molecules when the sample is not illuminated. To induce the patterns, the cell is irradiated with a frequency doubled Nd^+3^:YVO_4_ laser, with wavelength *λ*_0_ = 532 *nm* in the absorption band of the dopants, and with vertical polarization (following *y*-axis, cf. Fig. 1). The cell was subjected to input powers between *P* = 100 mW and *P* = 200 mW. Two plano-convex lenses increase the laser beam diameter to 2 *cm*. Additionally, two linear crossed polarizers *P*_1_ and *P*_2_ are positioned at the input and output of the dye-doped nematic liquid crystal sample, respectively, to analyze the response of the light that crosses the cell. Likewise, the second polarizer *P*_2_ (analyser) can be rotated with respect to the first polarizer *P*_1_ to characterise the birefringence properties of the liquid crystal sample. A beam-splitter (BS) is placed in between the liquid crystal sample and polarizer *P*_2_ to decrease the intensity of light, and thus, to achieve a better image. The transmitted beam is recorded with a CCD camera (Thorlabs DCU224M, 1280 × 1024 pixels).
